# Quality of life in patients with advanced gastric cancer: a randomized trial comparing docetaxel, cisplatin, 5-FU (TCF) with epirubicin, cisplatin, 5-FU (ECF)

**DOI:** 10.1186/1471-2407-6-274

**Published:** 2006-12-05

**Authors:** Sanambar Sadighi, Mohammad Ali Mohagheghi, Ali Montazeri, Zahra Sadighi

**Affiliations:** 1 Cancer Research Center, Cancer Institute, Imam Hospital, Tehran University of Medical Sciences, Tehran, Iran; 2 Iranian Institute for Health Sciences Research, Tehran, Iran

## Abstract

**Background:**

Health related quality of life (HRQOL) is an important outcome after treatment for upper gastrointestinal carcinoma. This study aimed to compare HRQOL in patients with advanced gastric cancer (GC) receiving either a standard or an experimental treatment.

**Methods:**

Seventy-one patients have been treated in Cancer Institute (Tehran, Iran) with docetaxel, cisplatin, 5 FU (TCF) or epirubicin, cisplatin, 5-FU (ECF) and were followed from Jan 2002 to Jan 2005. End points were response rate, HRQOL and survival. HRQOL was assessed using the EORCT QLQ-C30 at baseline and after the third cycle of chemotherapy.

**Results:**

The baseline HRQOL scores were comparable between two groups. After treatment improvement was seen in a number of items and domains except for cognitive functioning, and diarrhoea. Pain decreased and physical functioning improved in both groups. However, only the TCF group showed statistically and clinically meaningful improvement in global QOL (P = 0.001). Surgical and pathologic response was better with TCF but there was no difference in survival rate between two groups.

**Conclusion:**

Docetaxel based treatment (TCF) showed better palliation and improvement of global QOL as compared with epirubicin based treatment (ECF). However, it seems that regardless of treatment offered, effective chemotherapy was the most important factor affecting QOL in these patients.

## Background

When assessing the value of a particular anticancer treatment it is important to consider the impact it may have not only on length of survival but also on health related quality of life (HRQOL). In medical care of patients with gastric cancer, the 5-year survival is a crucial outcome. However, in the last few years there has been an increasing awareness about quality of survival. HRQOL especially is important for patients suffering from advanced gastric cancer, whose life expectancy may be short [1]. Despite the fact that numerous comparative therapeutic studies have been completed, there are relatively few reports of comparisons of the HRQOL effects of these differing therapies. Given that most patients with advanced gastric cancer are not cured and many regimens have similar efficacy, differences in HRQOL may help to determine which regimen is preferred [2]. As it has been suggested using quality of life assessment in clinical trials of patients with gastric cancer will help to define the role of potentially curative surgery, palliative treatments and adjuvant therapy [3]. A recent study demonstrated that quality of life might improve in patients with advanced gastric cancer, treated with second-line chemotherapy [4].

This article reports the results of HRQOL assessments obtained at baseline and after 3 cycles of chemotherapy in Iranian patients with advanced gastric adenocarcinoma enrolled in a randomized clinical trial of docetaxel, cisplatin, 5-FU versus epirubicin, cisplatin, 5-FU.

## Methods

### Design

Patients with histological confirmed gastric adenocarcinoma were entered into this randomized trial. Patients with primary or recurrent gastric adenocarcinoma (stage III or IV) were randomly assigned to receive three to six cycles of either ECF (epirubicin 60 mg/m^2^, cisplatin 60 mg/m^2 ^and 5-FU 750 mg/m^2^/day as 5 days continuous infusion) or TCF (docetaxel 60 mg/m^2^, cisplatin 60 mg/m^2 ^and 5-FU 750 mg/m^2 ^in the same dose and schedule of ECF) every 3 weeks. There were no industry funding, fees or salary for this protocol.

The primary objective of the study was to determine whether substitution of docetaxel for epirubicin in combination with cisplatin and 5-FU would improve response rate of advanced gastric cancer. Secondary endpoints were QOL, overall survival, and progression-free survival [5]. The Cancer Research Center review board approved the study and all patients signed the written informed consent dictated by the ethics committee of Tehran University of Medical Sciences.

### Quality of life assessment

QOL was assessed using the Iranian version of the European Organization for Research and Treatment of Cancer Quality of Life Questionnaire, EORTC QLQ-C30 [6]. It is one of the best-known cancer-specific questionnaires for measuring QOL in cancer patients [7]. The questionnaire is consisting of 30 items assessing five functional domains (physical, role, emotional cognitive, and social), symptom scales (fatigue, nausea and pain) and six single items (dyspnoea, sleep disturbances, appetite loss, constipation, diarrhoea, and financial impact of the disease and treatment), and a single global QOL scale. Quality of life was assessed before the first cycle of chemotherapy, and within one month after the third cycle of chemotherapy.

### Statistical analysis

For comparing patients' characteristics in two groups t-test or chi-square were used. The QLQ-C30 responses were scored and analyzed according to the scoring manual provided by the EORTC Study Group on Quality of Life [8]. First, the mean baseline scores for each treatment groups were calculated. Then, after treatment, the mean change score from baseline was calculated for all patients and compared between the two treatment groups. Two-related sample t-test (paired samples t-test) was used for statistical comparison. Survival analysis was performed using the Kaplan-Meier test.

## Results

Between January 2002 and January 2005, 86 patients were randomized into the two study groups. Objective response rate (more than 50% decreases in tumor size) was seen in 37% and 42% of ECF and TCF group respectively. Although surgery results were better in the TCF group (P = 0.01), we did not find statistically significant differences in clinical response to chemotherapy (Table 1).

For HRQOL evaluation, only 71 patients were included in the comparative analysis because 15 patients did not complete the QOL measurements at the beginning of the study. Table 1 shows demographic and clinical characteristics of the study sample, as well as the 71 participants in the QOL assessment by the treatment arms. They were quite similar. Most patients underwent a laparotomy before the study and had measurable residual or metastatic disease. The most frequent sites of disease at the time of random assignment were peritoneum, liver and paraaortic lymph nodes in both groups. Compliance with QOL questionnaire completion was excellent. Less than 4% of items had missing data.

The baseline quality of life scores for each study arms are shown in Table 2. At baseline and before chemotherapy patients in both groups showed impairment of global quality of life and most of functioning scales except for cognitive functioning. The baseline QOL scores were comparable between two groups, with the exception of emotional functioning and a better global QOL in the ECF group.

Table 3 lists the mean score changes from baseline to post treatment by the two study arms. Generally both groups showed improvements compared with their baseline status in a number of domains and items except for cognitive functioning, diarrhoea and financial aspect of disease and treatment that showed deterioration. Although in both groups physical functioning improved and pain decreased but only in the TCF group statistically and clinically significant improvement was observed for global QOL (P = 0.001), social (P = 0.03) and emotional functioning (P = 0.004), pain (P = 0.03) and sleep difficulties (P = 0.02).

To examine whether the observed differences were significant, comparisons were made and the results are shown in Table 4. As indicated there were no significant differences between two groups after treatment except for social functioning (P = 0.03) and nausea and vomiting (P = 0.01) in the TCF group.

**Figure 1:**
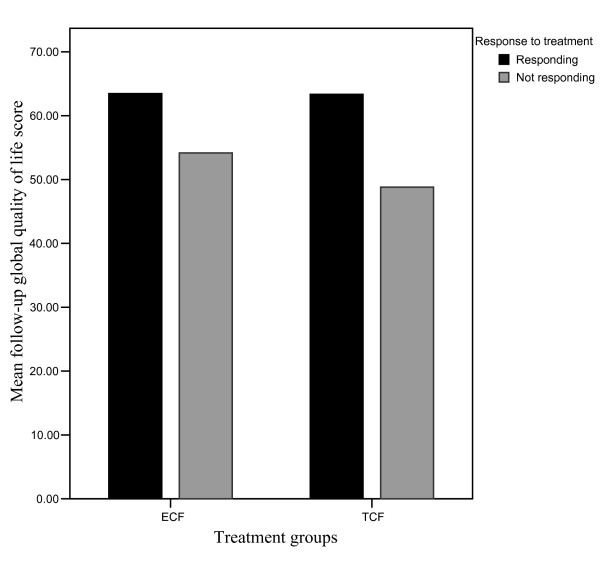
Follow-up global quality of life scores in responding and not responding patients for each treatment group (Higher values show better global quality of life): ECF: Mean score (SD); Responding 63.5 (14.2), Not responding 54.2 (14.9). TCF: Mean score (SD); Responding 63.3 (16.6), Not responding 48.8 (13.5).

Patients with proven tumor regression most frequently had an improvement of HRQOL domains (by example the follow-up global quality of life scores for responding and not responding patients to chemotherapy is shown in Figure 1. However, the median survival time for both groups was 12 months with a 95% confidence interval of 8 to 14 and 7 to 17 months for the ECF and TCF groups respectively.

## Discussion

This trial was undertaken primarily to determine if TCF offered superior response rate compared with a standard ECF treatment. QOL measures were assessed to provide patient-reported symptoms, both disease and treatment related, as well as providing a general assessment of global well-being and functional status of patients treated with these chemotherapy regimens. The study demonstrated a better response with the TCF group (although not significant) and very similar survival between two regimens [5].

Based on our data the TCF regimen was associated with a meaningful improvement in health related QOL. Although overall about 40% of the patients had major response to chemotherapy [5], the study findings suggest that improvement of HRQOL was due to palliative effect of chemotherapy. The lack of apparent impact of toxicity on global functioning of responding patients is clinically useful data that may assist both the patient and the physician in the discussion about the anticipated effects of the proposed chemotherapy treatment.

The EORTC QLQ-C30 was sensitive to detect HRQOL issues that were important to patients within our specific treatment groups. To identify aspects of HRQOL in which the patients clearly had gained meaningful improvement, a significant change of 5 points and p value less than 0.03 was considered to be important [9]. Accordingly one might argue that patients in the TCF group experienced a better QOL after their treatments. For example the TCF group shoed 9.7 points change in their baseline global quality of life while the same figure for the ECF group was 2.4 points.

As in any HRQOL analysis, the data need to be interpreted with caution bearing in mind the study limitations. This study represents QOL information for 71 patients (80% of patients who took part in the study), although their baseline characteristics were comparable with the entire patients. In addition, the baseline QOL scores and symptoms in some patients may have been due to the effect of surgery (laparotomy) rather than gastric cancer itself. Thus, improvement in QOL and symptoms may have occurred even in the absence of chemotherapy. However, one should consider that HRQOL improvement was confined only to the responders and were not seen in patients who experienced progression of the disease. There is evidence that the type of surgery does not significantly influence the quality of life in patients undergoing curative surgery for gastric cancer [10].

Unfortunately at the time of current project the Iranian version of the FACT-G (a well known general cancer specific quality of life questionnaire) or EORTC QLQ-STO22, a site-specific measure of quality of life in patients with gastric cancer were not available to use in this study. Therefore it is important that in the future studies at least to supplement the QLQ-C30 with the QLQ-STO22 in measuring quality of life in patients with gastric cancer undergoing surgery, surgery and chemo-radiotherapy, palliative chemotherapy, palliative surgery and best supportive care [11]. As suggested QOL in patients with gastric cancer deserves more systematic studies, especially as one of the outcome measures in randomized clinical trials [1].

## Conclusion

In conclusion, QOL measures provide helpful information of patient-reported symptoms that is essential for full evaluation of new treatments for patients with gastric cancer. The study findings indicated that the group treated with TCF enjoyed more improvement with regard to global QOL as compared with the group that treated with ECF. However, it seems that regardless of treatment offered, effective chemotherapy was the most important factor affecting QOL in these patients.

## Competing interests

The author(s) declare that they have no competing interests.

## Authors' contributions

SS was the main investigator, designer and executive manager of the clinical trial. MAM participated in the design of the study and carried out all of the patients operations. AM participated in the design and acquisition of QOL data and performed the statistical analysis. ZS participated in the sequence alignment and coordination. All authors read and approved the final manuscript.

## Pre-publication history

The pre-publication history for this paper can be accessed here:



## Figures and Tables

**Table 1:** The characteristics of patients in each treatment arms

	**ECF group^1^**		**TCF group^2^**		**P^4^**
	All (n = 42) No. (%)	QOL^3 ^(n = 35) No. (%)	All (n = 44) No. (%)	QOL^3 ^(n = 36) No. (%)	

**Age**					0.23
Mean (SD)	57. 2 (9.83)	58.1 (10.48)	55.4 (14.04)	54.2 (16.18)	
**Gender**					0.20
Male	34 (81)	28 (80)	31 (70)	24 (67)	
Female	8 (19)	7 (20)	13 (30)	12 (33)	
**Tumor size**					0.48
T2	8 (19)	8 (23)	5 (11)	5 (14)	
T3	15 (36)	12 (34)	15 (34)	11 (31)	
T4	19 (45)	15 (43)	24 (55)	20 (55)	
**Lymph node**					0.39
L1	20 (48)	18 (51)	14 (32)	14 (39)	
L2	17 (40)	13 (37)	20 (45)	14 (39)	
Not involvement	5 (12)	4 (12)	10 (23)	8 (22)	
**Differentiation**					0.30
Well differentiated	7 (17)	7 (20)	5 (11)	4 (11)	
Others	35 (83)	28 (80)	39 (89)	32 (89)	
**Tumor of cardia**					0.56
Yes	19 (45)	14 (40)	15 (34)	12 (33)	
Others	23 (55)	21 (60)	29 (66)	24 (67)	
**Metastatic site**					0.92
Liver	7 (17)	7 (20)	9 (20)	7 (20)	
Peritoneum	13 (31)	10 (29)	14 (32)	9 (25)	
Para aortic	5 (12)	4 (11)	3 (7)	3 (8)	
Multiple sites	17 (40)	14 (40)	18 (41)	17 (47)	
**Primary disease**					0.93
Yes	30 (71)	24 (69)	33 (75)	25 (69)	
No (recurrent)	12 (29)	11 (31)	11 (25)	11 (31)	
**Response to chemotherapy**					0.69
Responding (complete and partial)	17 (40)	13 (37)	18 (41)	15 (42)	
Not responding (stable or progressive disease)	25 (60)	22 (63)	26 (59)	21 (58)	

^1 ^Epirubicin, cisplatin and 5-FU

^2 ^Docetaxel, cisplatin and 5-FU

^3 ^Those who completed the quality of life questionnaire

^4 ^P values derived from t-test or chi-square analyses for comparison between two groups of patients who completed the quality of life questionnaire

**Table 2:** Quality of life baseline scores as measured by the EORTC QLQ-C30 for each study arms

	**ECF group**	**TCF group**	**P**
	Mean (SD)	Mean (SD)	

Functioning scores*			
Physical functioning	65.3 (13.4)	66.1 (11.1)	0.79
Role functioning	68.5 (15.5)	68.0 (18.4)	0.89
Emotional functioning	64.0 (12.1)	56.6 (14.7)	0.02
Cognitive functioning	85.7 (10.8)	84.2 (14.3)	0.61
Social functioning	68.1 (13.6)	68.5 (13.1)	0.89
Global quality of life	55.2 (17.4)	45.1 (14.4)	0.01
Symptom scores**			
Fatigue	43.5 (14.8)	49.6 (17.3)	0.11
Nausea and vomiting	22.8 (19.8)	33.8 (23.7)	0.04
Pain	40.0 (16.7)	47.1 (21.2)	0.12
Dyspnoea	7.62 (16.3)	12.0 (21.3)	0.33
Sleep difficulties	31.4 (25.5)	40.7 (21.2)	0.09
Appetite loss	39.0 (22.1)	46.3 (24.3)	0.19
Constipation	14.3 (21.8)	14.8 (26.9)	0.93
Diarrhoea	3.81 (10.7)	9.25 (18.8)	0.14
Financial difficulties	52.4 (20.2)	44.4 (21.1)	0.11

* Higher scores show better functioning and global quality of life.

** Higher scores indicate greater symptoms.

**Table 3:** Quality of life mean score changes before and after 3 cycles of chemotherapy (follow-up scores minus baseline scores) for each study arms

	**ECF group**		**TCF group**	
**Functioning scores^1^**	Mean (SD) score changes	P^3^	Mean (SD) score changes	P^3^

Physical functioning	4.1 (13.6)	0.08	2.3 (14.8)	0.36
Role functioning	0.57 (14.3)	0.81	2.7 (18.9)	0.38
Emotional functioning	-0.06 (8.3)	0.96	8.0 (15.4)	0.004
Cognitive functioning	-2.5 (13.4)	0.27	-6.1 (17.0)	0.04
Social functioning	-2.3 (14.6)	0.36	5.2 (14.1)	0.03
Global quality of life	2.4 (14.5)	0.34	9.7 (16.8)	0.001
**Symptom scores^2^**				
Fatigue	-0.99 (14.8)	0.69	-4.6 (14.1)	0.05
Nausea and vomiting	-3.5 (19.6)	0.29	-1.4 (29.9)	0.78
Pain	-6.3 (14.2)	0.01	-5.7 (15.1)	0.03
Dyspnoea	-2.6 (14.9)	0.31	-0.92 (20.3)	0.78
Sleep difficulties	-5.8 (23.5)	0.15	-11.3 (27.5)	0.02
Appetite loss	-4.6 (19.8)	0.17	-5.4 (29.3)	0.27
Constipation	-1.1 (29.4)	0.83	0.92 (36.9)	0.88
Diarrhoea	9.8 (19.0)	0.004	0.32 (27.6)	0.94
Financial difficulties	9.1 (23.6)	0.03	11.6 (19.3)	0.001

^1 ^For functioning scores positive values show improvements and negative values indicate deteriorations.

^2 ^For symptom scores negative values show improvements and positive values indicate deteriorations.

^3 ^P values derived from the paired t-tests.

SD = Standard deviation

**Table 4:** Comparison of quality of life scores between two groups after treatment

	**ECF group**	**TCF group**	
**Functioning scores^1^**	Mean (SD) score	Mean (SD) score	P^3^

Physical functioning	69.4 (14.9)	68.3 (19.1)	0.81
Role functioning	69.1 (16.7)	70.8 (21.2)	0.71
Emotional functioning	63.9 (12.5)	64.6 (13.5)	0.82
Cognitive functioning	83. 2 (11.4)	78.1 (13.1)	0.08
Social functioning	65. 8(15.6)	73.7 (15.5)	0.03
Global quality of life	57.6 (15.1)	54.8 (16.3)	0.46
**Symptom scores^2^**			
Fatigue	42.5 (12.7)	44.9 (16.7)	0.48
Nausea and vomiting	19.3 (16.7)	32.4 (26.1)	0.01
Pain	32.8 (17.1)	41.4 (22.6)	0.08
Dyspnoea	7.6 (16.3)	11.1 (21.1)	0.13
Sleep difficulties	25.5 (21.4)	29.4 (22.2)	0.45
Appetite loss	34.4 (24.9)	40.8 (27.7)	0.31
Constipation	13.2 (21.2)	15.7 (25.8)	0.65
Diarrhoea	13.6 (19.9)	9.5 (17.0)	0.35
Financial difficulties	61.4 (25.7)	56.0 (24.8)	0.37

^1 ^Higher scores show better functioning and global quality of life.

^2 ^Higher scores indicate greater symptoms.

^3 ^P values derived from independent samples t-tests.

SD = Standard deviation
